# Two Ways of Looking at an Oyster

**Published:** 1903-01-10

**Authors:** 


### EDITOR'S LETTER-BOX.

[Our Correspondents are reminded that prolixity is a great bar to publication, and that brevity of style and conciseness of statement greatly facilitate early insertion.]

### TWO WAYS OF LOOKING AT AN OYSTER.

We have received from Mr. George Tabor a long letter in the course of which he says:—If the discharge of crude sewage is continued in our estuaries and seas, demands will go on increasing for the close of first one class of fishery then another, and so on, until in course of time all will be closed, and the fishing industry ruined. And even then danger to public health would still arise in other ways from the sewage-polluted districts. If the discharge of crude sewage was absolutely necessary in the public interest, the question would to some degree be altered. But it is well known that the discharge of crude sewage is not necessary. There are many ways in which the sewage can be employed to far more profitable ends than destroying our fisheries. Moreover, it can be treated and rendered practically innocuous. The expense of a series of bacterial tanks interposed between the final junction of sewers and the sea would in the majority of cases be very small.

The Government recognise that this is the true remedy for protecting public health. Because the "Oyster Bill of 1899," which, of course, was in the interest of public health, was withdrawn because it was clearly proved to the Select Committee that considered it that the danger to public health was caused not so much by the oyster as by the sewage which polluted it. And since the date of that withdrawal nearly all new sewage schemes have been submitted to the Sea Fisheries Committees in their respective districts before being approved. And where fishery interests have been threatened the schemes have had to be altered to meet the requirements of the Fishery Committees. I mention these facts to prove that the oyster trade is not alone in their contention, and that its attitude is not in the least unreasonable. The root of the matter is the compulsory treatment of all sewage before its discharge into the sea. The demand is perfectly practicable, because it is done with all new sewage schemes, and can easily be applied to most of the old systems. And not only will the health of the public be thus efficiently safeguarded, but the fisheries will be preserved as a valuable national food supply.

